# Consumer Confidence in the Responsible Use of Digital Health Data After the COVID-19 Pandemic

**DOI:** 10.1001/jamanetworkopen.2024.61907

**Published:** 2025-02-26

**Authors:** Ravi Gupta, Meghana Sharma, Nandita Mitra, Carolyn C. Cannuscio, Raina M. Merchant, David A. Asch, David Grande

**Affiliations:** 1 Division of General Internal Medicine, Johns Hopkins University School of Medicine, Baltimore, Maryland; 2 Department of Health Policy and Management, Bloomberg School of Public Health, Johns Hopkins University, Baltimore, Maryland; 3 University of Illinois College of Medicine, Chicago; 4 Department of Biostatistics, Epidemiology and Informatics, University of Pennsylvania, Philadelphia; 5 Department of Family Medicine and Community Health, University of Pennsylvania, Philadelphia; 6 Leonard Davis Institute of Health Economics, University of Pennsylvania, Philadelphia; 7 Department of Emergency Medicine, University of Pennsylvania, Philadelphia; 8 Division of General Internal Medicine, Department of Medicine, Perelman School of Medicine, University of Pennsylvania, Philadelphia

## Abstract

This study compares 2020 and 2022 surveys to identify changes in how respondents view use of their health information by public and private organizations, including by political leaning.

## Introduction

Health care data use and protection are largely regulated by existing law, but no comparable set of rules regulates the use of data generated by consumers through non–health care sources (eg, wearable devices, smartphone applications).^1,2^ In a 2020 survey, we found the US population had greatest confidence in clinical organizations and public institutions when compared with digital technology and health care companies to responsibly use their digital health data.^3^ Given growing mistrust in public and health care institutions after the COVID-19 pandemic and increasing political polarization on public health issues,^4^ we hypothesized a change in confidence in institutions to responsibly use health data, particularly by political ideology.

## Methods

In this survey study, we administered a national survey in English/Spanish in June 2022 and compared average responses with those of a survey conducted in July 2020.^3^ The survey instrument is available in eMethods of Supplement 1. Survey respondents were sampled from a web-enabled research panel representative of the US population (Ipsos KnowledgePanel).^5^ Hispanic and African American respondents were oversampled. This study was considered exempt by the institutional review board at the University of Pennsylvania based on the minimal risk of the research and use of deidentified data. This study followed reporting guidelines and ethical conduct of public opinion and survey research per the AAPOR. Participants provided informed consent.

In both survey years, respondents rated their confidence in whether each of 16 public and private organizations would use their digital health data responsibly. The web-enabled panel provided self-reported sociodemographic data, including race and ethnicity, for respondents. Additional survey items measured political ideology.

We used poststratification weights to account for oversampling and allow US adult population representativeness. For both 2020 and 2022, we used separate multivariable logistic regression models to estimate associations between political ideology and other respondent characteristics and confidence in the responsible use of digital health information. We used multiple imputation to account for incomplete responses. Statistical significance was set at 2-sided *P* < .05. Analyses were conducted in Stata version 18.0 (StataCorp).

## Results

In 2022, of 3194 respondents contacted, 1851 (58%) responded (954 women [51.5%]; mean [SD] age, 48.1 [17.9] years), most of whom were at least somewhat confident in physician offices, university hospitals, the Centers for Disease Control and Prevention (CDC), and the National Institutes of Health (NIH) (Figure). Compared with those in 2020, respondents in 2022 expressed lower confidence in the CDC (−4.3% [95% CI, −7.4% to 1.2%]), the NIH (−2.6% [95% CI, −5.6% to 0.4%]), and local public health departments (−1% [95% CI, −4.1% to 2.1%]) and higher confidence in the federal government (6.1% [95% CI, 3.2% to 9.0%]), FitBit (2.3% [95% CI, 0.1% to 5.3%]), and genetic testing companies (2.2% [95% CI, −0.5% to 5.7%]).

**Figure.**
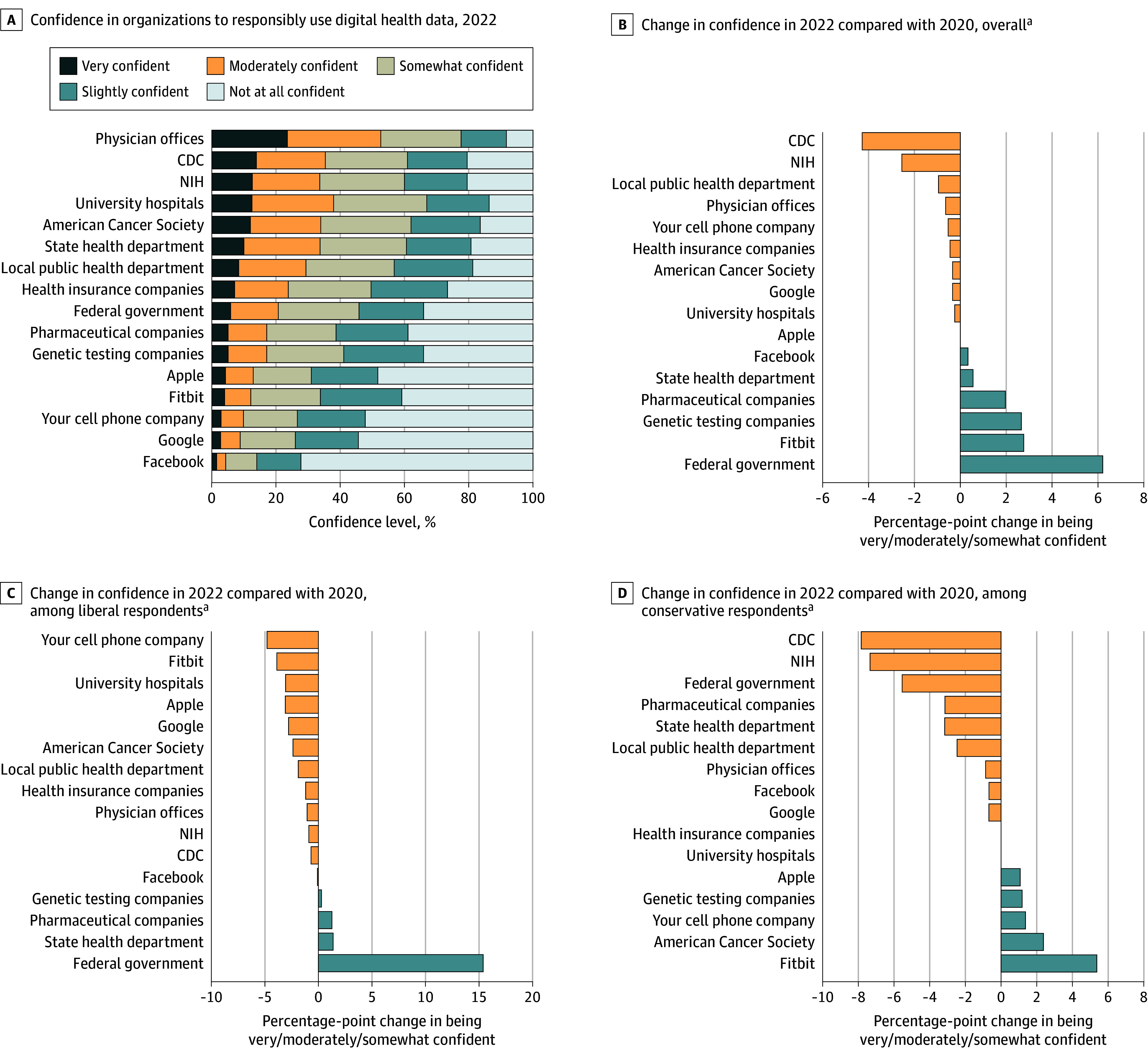
Confidence in Organizations to Responsibly Use Digital Health Information Consumer confidence in each organization to treat consumers’ digital health data responsibly in 2022 (A). Change in consumer confidence in organizations to use digital health information in 2022 relative to 2020 overall (B) and among liberal (C) and conservative (D) respondents. Weighted distribution of responses to 16 survey questions evaluating confidence in public and private organizations to use digital health information responsibly administered in 2022 and 2020. In both years, respondents were asked, “We are going to name some institutions, companies, and organizations that might collect and use digital health information from you. How confident are you that they will use your digital health information responsibly?” CDC indicates Centers for Disease Control and Prevention; NIH, National Institutes of Health. ^a^Data and analyses from the 2020 survey were published in a prior study.^3^

In adjusted models, in both 2020 and 2022, politically liberal respondents were most confident in physician offices (84% [95% CI, 81%-86%] vs 83% [95% CI, 80%-86%]), the CDC (80% [95% CI, 77%-82%] vs 79% [95% CI, 75%-82%]), and the NIH (77% [95% CI, 74%-80%] vs 75% [95% CI, 71%-79%]) and least confident in Facebook (14% [95% CI, 12%-17%] vs 13% [95% CI, 10%-16%]) and cell phone companies (28% [95% CI, 25%-31%] vs 23% [95% CI, 19%-27%]) (Table). In both years, conservative respondents were most confident in physician offices (74% [95% CI, 71%-77%] vs 73% [95% CI, 69%-77%]) and university hospitals (58% [95% CI, 55%-62%] vs 59% [95% CI, 54%-63%]) and least confident in Facebook (12% [95% CI, 10%-14%] vs 12% [95% CI, 9%-15%]) and Google (22% [95% CI, 19%-25%] vs 22% [95% CI, 18%-25%]). The greatest change in confidence in 2022 relative to 2020 among liberal and moderate respondents was in the federal government, increasing by 13% (95% CI, 8%-19%) for liberal and 9% (95% CI, 4%-14%) for moderate respondents. Among conservative respondents, the greatest change in confidence was in the CDC (−10% [95% CI, −15% to −4%]), NIH (−8% [95% CI, −14% to −3%]), and federal government (−7% [95% CI, −12% to −2%]).

**Table.** Confidence in Public and Private Organizations by Political Ideology, 2020 and 2022^a^^,^^b^

Organization	Political ideology, adjusted probability, % (95% CI)											
	Overall			Liberal			Moderate			Conservative		
	2020	2022	*P* value	2020	2022	*P* value	2020	2022	*P* value	2020	2022	*P* value
Health care organizations												
University hospitals	67 (65-69)	67 (65-69)	.98	77 (74-80)	73 (69-77)	.12	67 (64-70)	69 (65-73)	.43	58 (55-62)	59 (54-63)	.73
Physician offices	78 (76-79)	78 (76-80)	.89	84 (81-86)	83 (80-86)	.62	76 (74-79)	78 (74-81)	.36	74 (71-77)	73 (69-77)	.70
American Cancer Society	62 (61-64)	62 (60-65)	.95	72 (69-75)	68 (63-72)	.15	63 (60-66)	63 (59-67)	.97	54 (50-57)	56 (52-60)	.46
Health care companies												
Genetic testing companies	39 (37-41)	41 (39-44)	.20	42 (38-45)	43 (38-47)	.73	38 (35-41)	43 (39-47)	.05	38 (35-41)	37 (33-42)	.72
Pharmaceutical companies	37 (35-39)	39 (37-41)	.19	36 (32-39)	37 (33-42)	.73	37 (34-40)	43 (39-47)	.02	38 (35-41)	35 (31-39)	.24
Health insurance companies	50 (48-52)	50 (47-52)	.84	49 (46-53)	47 (43-51)	.46	52 (49-55)	52 (47-56)	.97	49 (46-52)	49 (45-54)	.97
Technology companies												
Google	26 (25-28)	26 (24-28)	.96	29 (26-32)	26 (22-30)	.24	28 (26-31)	30 (26-33)	.36	22 (19-25)	22 (18-25)	.97
Apple	31 (29-33)	31 (29-33)	.93	33 (30-36)	31 (27-35)	.43	32 (29-35)	33 (29-37)	.70	28 (25-31)	28 (25-32)	.97
Facebook	14 (13-15)	14 (13-16)	.59	14 (12-17)	13 (10-16)	.62	15 (13-17)	16 (13-19)	.59	12 (10-14)	12 (9-15)	.96
Fitbit	31 (29-32)	34 (32-37)	.02	34 (30-37)	31 (27-35)	.27	31 (28-34)	37 (33-41)	.02	28 (25-31)	34 (30-38)	.02
Your cell phone company	27 (25-28)	27 (25-29)	.95	28 (25-31)	23 (19-27)	.05	27 (25-30)	29 (26-33)	.36	25 (23-28)	27 (23-30)	.36
Government												
Federal government	41 (39-42)	46 (43-48)	<.001	45 (41-48)	58 (54-63)	<.001	41 (38-44)	50 (46-54)	<.001	36 (33-39)	30 (26-34)	.02
CDC	65 (64-67)	61 (58-63)	.002	80 (77-82)	79 (75-82)	.65	67 (64-70)	63 (59-66)	.12	52 (49-55)	42 (38-47)	<.001
NIH	63 (61-64)	60 (58-62)	.04	77 (74-80)	75 (71-79)	.43	62 (59-65)	63 (59-67)	.67	51 (48-55)	43 (39-47)	.003
Local public health department	58 (56-60)	57 (54-59)	.56	69 (66-73)	67 (63-71)	.46	56 (53-59)	58 (54-62)	.43	49 (46-53)	46 (41-50)	.30
State health department	60 (58-62)	61 (58-63)	.72	71 (68-75)	71 (67-76)	.97	60 (57-63)	63 (59-67)	.24	50 (47-53)	48 (44-52)	.43

Abbreviations: CDC, Centers for Disease Control and Prevention; NIH, National Institutes of Health.

^a^ Among those who responded, we accounted for missing data (confidence in each organization, political ideology, health status) by using multiple imputation by chained equations (MICE) with the following model for 20 imputations: year (2020 or 2022) + age + Hispanic/not Hispanic + education + household income + household ownership status + number of adults (>18 y old) in household + metropolitan status + US state. All missingness rates were <2%.

^b^ Adjusted probabilities from multivariable logistic regression models that included the following additional covariates: age, sex, race, ethnicity, household income, education, metropolitan statistical area, region, and health status (excellent/very good/good vs fair/poor). Percent column reflects adjusted percentages of respondents who reported being “somewhat,” “moderately,” or “very” confident (yes/no) in each respective institution to use their digital health information responsibly, stratified by political ideology.

## Discussion

In this study, confidence in organizations to use health data responsibly was largely unchanged from 2020 to 2022, but polarization increased between politically liberal and conservative respondents. Compared with 2020, in 2022 liberal respondents reported increased confidence in the federal government to use digital health data responsibly, while conservative respondents reported decreased confidence in the federal government and agencies. Changes in confidence may be related to the prominence of these agencies (CDC, NIH) during the COVID-19 pandemic, a 2020 national election with a change in the governing political party, and political polarization.^6^ Our study is limited in that we did not survey the same respondents in the 2 years but instead report averages from surveys in each year.

The public’s views and increasing political polarization regarding the use of digital health data are important to understand for advancing digital privacy legislation.

## Notes

Supplement 1. **eMethods.** Survey Instrument

Supplement 2.
Data Sharing Statement
