# Supplementary material for: Consumer Confidence in the Responsible Use of Digital Health Data After the COVID-19 Pandemic
Source: JAMA Netw Open. 2025 Feb 26;8(2):e2461907. doi: 10.1001/jamanetworkopen.2024.61907 (PMC11866024; doi:10.1001/jamanetworkopen.2024.61907)
Supplement: Supplement 1. — eMethods. Survey Instrument [file jamanetwopen-e2461907-s001.pdf]

## Supplemental Online Content

Gupta R, Sharma M, Mitra N, et al. Consumer confidence in the responsible use of digital health data after the COVID-19 pandemic. *JAMA Netw Open*. 2025;8(2):e2461907. doi:10.1001/jamanetworkopen.2024.61907

**eMethods.** Survey Instrument

This supplementary material has been provided by the authors give readers additional information about their work.

## eMethods. Survey Instrument

### Sample Variables

- KP standard demographics
- xspanish
- xhispan
- xacslang

### Quota Description

### Main Questionnaire (including screener, if applicable)

*Programming Notes:*
- *Code all refusals as -1.*
- *Use default instruction text for each question type unless otherwise specified.*
- *Do not prompt on all questions. (Remove this instruction if sample is all opt-in, client list sample, or otherwise not KP.)*

### Main Survey

Base: All respondents

**Intro [Display]**
This survey is being conducted by the University of Pennsylvania. We want to learn your feelings about how digital information is collected and used. The questions should only take 15 minutes. All of your responses will remain completely confidential.  Let's get started.

Please note that this survey may include some personal questions about your health or political opinions. This information can help us select surveys that might be more relevant to you and improve analyses for each study. Your responses are used for research purposes only and will remain anonymous – results are reported only for groups, not for individuals. Participation is voluntary, and you can choose not to answer any question. Answering the questions means that you accept us collecting the data.

*Scripting note: For each respondent, randomly select 15 rows from the design tab in "Conjoint1_Design.xlsx", insert text in conjoint based on values for each column within the each row. loop Conjoint1_x 15 times, each iteration with an insertion pattern based on lookup values in "Conjoint1_Design.xlsx". RANDOMIZE ORDER OF THE 15 QUESTIONS AND RECORD*

Base: All respondents

**Conjoint1_Intro [Display]**
**INTRODUCTION**
Most Americans use digital technology in their daily lives. When you use digital technology, like your cell phone or computer, you leave information about yourself online. Experts believe that most of the information you leave behind can reveal something about your health. It is often possible to identify who you are from the information you leave behind.

This health information has a lot of possible uses. We are interested in what you think about these

uses.

You will be shown **15 possible uses** of your digital information. In each case, you will be shown **who** will use the information, **what** information will be used, and **why** they are using it. Please indicate how willing you would be to share your health information for each situation.

**Because each situation is different, please read each one carefully before providing your answer.** We appreciate your honest opinions.

Base: All respondents

**Conjoint1_x [Conjoint]**

Please imagine you were asked to share your information in the following situation:

***Who will use your information***: *[INSERT USER VALUE BASED Lookup "Conjoint1_Design.xlsx" with insertion texts in "Insertion_Reference" tab]*
***What information will be used***: *[INSERT INFO VALUE BASED Lookup "Conjoint1_Design.xlsx" with insertion texts in "Insertion_Reference" tab]*
***What they will use it for***: *[INSERT USE VALUE BASED Lookup "Conjoint1_Design.xlsx" with insertion texts in "Insertion_Reference" tab] [INSERT DISEASE VALUE BASED Lookup "Conjoint1_Design.xlsx" with insertion texts in "Insertion_Reference" tab]*

How likely would you be to share your digital information in this situation?

1. Definitely WOULD share
2. Probably WOULD share
3. Unsure
4. Probably WOULD NOT share
5. Definitely WOULD NOT share

Base: All respondents

**Q1 [S]**

In general, how would you rate your health?

1. Excellent
2. Very good
3. Good
4. Fair
5. Poor

Base: All respondents

**Q2 [S]**

In the past 2 years, have you been the victim of identity theft, credit card theft or had other information stolen on the internet?

1. Yes
2. No
3. Not sure

NO Q3

Base: All respondents RANDOMIZE AND RECORD

**Q4 [Grid]**

Next, we are going to name some institutions, companies and organizations that might collect and use digital health information from you. How confident are you that they will use your digital health information responsibly?

*Scripting note: Split onto 2 screens one with 9 items and one with 8 items.*

Statement in rows:

1. University hospitals
2. Doctors' offices
3. Genetic testing companies that you can pay to learn about your ancestry and genetic conditions
4. Pharmaceutical companies
5. Health insurance companies
6. Google
7. Apple
8. Facebook
9. FitBit
10. Your cell phone company
11. American Cancer Society
12. Local public health department run by your county or town
13. Your state health department
14. Federal government
15. Centers for Disease Control and Prevention (CDC)
16. National Institutes of Health (NIH)
17. Your local public library

Answer in columns:

1. Very confident
2. Moderately confident
3. Somewhat confident
4. Slightly confident
5. Not at all confident

Base: All respondents

Q5. [S]

Last week, did you work for pay at a job (or business)?

1. Yes
2. No - Did not work (or retired)

Base: All respondents

Q6. [S]

Currently, how often are you working full days at home?

1. Never
2. About once or twice per month
3. About once or twice per week
4. More than twice per week but not always
5. Always

Base: All respondents

Q7. [Grid]

In general, how much would you trust information regarding vaccines accessed through the following sources:

*Scripting note: Split onto 2 screens one with 6 items and one with 5 items.*

*Statements in rows: randomize order*

A. University hospitals
B. Doctors' offices
C. Pharmaceutical companies
D. Health insurance companies
E. People I follow on social media (e.g., TikTok, Twitter, Facebook)
F. Local public health department run by your county or town
G. Your state health department
H. Federal government
I. Centers for Disease Control and Prevention (CDC)
J. National Institutes of Health (NIH)
K. Your public library

*Answers in columns:*

1. A great deal
2. Considerably
3. Moderately
4. Slightly

5. Not at all

Base: All respondents

Q8_Intro1. [DISP]

Now we will ask your opinions about a new technology product that is under development to understand if the product would be useful to you.

*[Programming instructions: Randomize respondents to introduction text (3 options: A. neutral, B. soft privacy prime, C. strong privacy prime). Then randomize to one technology example (out of 4 options).]*

| RANDOMIZE 3 OPTIONS OF INTRO TEXT | INTRO TEXT FOR EACH OPTION |
|---|---|
| Option A:<br>*Neutral* | Digital technologies like smartphone apps can offer many opportunities for consumers to track and improve their health. Health apps range from tracking fitness goals to managing disease symptoms, and providing valuable information about personal health. The number of health apps and technology has increased in recent years. |
| Option B:<br>*Soft Privacy Prime* | Digital technologies like smartphone apps can offer many opportunities for consumers to track and improve their health. Health apps range from tracking fitness goals to managing disease symptoms, and providing valuable information about personal health. Although uses can be beneficial, some consumers have concerns surrounding the privacy of their data. However, some feel the benefits are worth the possible loss of privacy. |
| Option C:<br>*Strong Privacy Prime* | Digital technologies like smartphone apps can offer many opportunities for consumers to track and improve their health. Health apps range from tracking fitness goals to managing disease symptoms, and providing valuable information about personal health. Although uses can be beneficial, some consumers have concerns surrounding the privacy of their data. Some companies have been found to misuse user data and some sell it to third-party companies. However, some feel the benefits are worth the possible loss of privacy. |

COMPUTE DOV_INTROTEXT = 0.
IF SHOWN INTRO OPTION A, DOV_INTROTEXT = 1. IF SHOWN INTRO OPTION B, DOV_INTROTEXT = 2. IF SHOWN INTRO OPTION C, DOV_INTROTEXT = 3.

ON SAME SCREEN AS Q8Intro_2

Base: All respondents

Q8Intro_2. [DISP]

**Please consider the following new technology product.**

| RANDOMIZE 4 TECHNOLOGY OPTIONS | TEXT FOR EACH TECHNOLOGY OPTION |
|---|---|
| *Tech Option A.* | A hospital has partnered with digital technology experts to create a new device that can help you improve your sleep so you feel more rested and energetic during the daytime. You would wear a small bracelet or ring when you sleep at night which would share information such as your bedtime and wake up time with your smartphone. An app on your smartphone would evaluate your sleep patterns and make suggestions for improvements. |
| *Tech Option B.* | A hospital has partnered with digital technology experts to create a new smartphone app that can help you improve your mood. The app will analyze your technology usage and social communication to determine if your mood is down or low. If the app detects signs that your mood is down or depressed, it will notify you and make suggestions on steps you can take to improve your mood. |
| Tech Option C. | A hospital has partnered with digital technology experts to create a new smartphone app that can detect falls and accidents. The app analyzes movement and GPS location from your smartphone. If it detects a fall or accidents, it will notify emergency services and your emergency contacts to get help. |
| *Tech Option D.* | A hospital has partnered with digital technology experts to create a new device that can monitor your vital signs including heart rate and oxygen levels. The wearable device collects and analyzes your vital signs throughout the day and will alert you if any irregularities are found such as a heart rate becoming too high or low. |

COMPUTE DOV_TECHTEXT = 0.
IF SHOWN TECH OPTION A, DOV_TECHTEXT = 1. IF SHOWN TECH OPTION B, DOV_TECHTEXT = 2. IF SHOWN TECH OPTION C, DOV_TECHTEXT = 3. IF SHOWN TECH OPTION D, DOV_TECHTEXT = 4.

Base: All respondents

Q8_1. [S]

How useful would this product be to you?

Note: click **here** to see the description of the product again. [IF RESPONDENT CLICKS "HERE" DISPLAY SPECIFIC DOV_TECHTEXT SEEN BY RESPONDENT]

1. Very useful
2. Moderately useful
3. Somewhat useful
4. Slightly useful
5. Not at all useful

Base: All respondents

Q8_2. [S]

How useful would this product be for a family member?

Note: click **here** to see the description of the product again. [IF RESPONDENT CLICKS "HERE" DISPLAY SPECIFIC DOV_TECHTEXT SEEN BY RESPONDENT]

1. Very useful
2. Moderately useful
3. Somewhat useful
4. Slightly useful
5. Not at all useful

Base: All respondents

Q8_3. [S]

How likely would you be to download this today if it were available for free?

Note: click **here** to see the description of the product again. [IF RESPONDENT CLICKS "HERE" DISPLAY SPECIFIC DOV_TECHTEXT SEEN BY RESPONDENT]

1. Very likely
2. Moderately likely
3. Somewhat likely
4. Slightly likely
5. Not at all likely

Base: All respondents

Q8_4. [S]

How concerned are you about the privacy of information the company would collect about you or a family member when you use this product?

Note: click **here** to see the description of the product again. [IF RESPONDENT CLICKS "HERE" DISPLAY SPECIFIC DOV_TECHTEXT SEEN BY RESPONDENT]

1. Very concerned
2. Moderately concerned
3. Somewhat concerned
4. Slightly concerned
5. Not at all concerned

Base: All respondents

Q9_intro. [Disp]

Next we are going to ask you about privacy protections that may or may not be important to you in deciding whether you would want to use this app.

We will show you some options to protect consumer privacy. You have a budget of 100 points to assign to the privacy protections. The more important a protection is to you, the more points it should receive. The total points allocated across the options should equal 100.

Base: All respondents

Q9_1. [Numeric] [Prompt once if responses do not equal 100]

Please allocate 100 points across the following protections. The more important a protection is to you, the more points it should receive.

**The company will:**

*Scripter: display box for each option, with active sum in the Total row; randomize options*

1. Tell you *how they use* your data. ____
2. Tell you *if they share* your data with third parties. ____
3. Be restricted in how they can use or store your data. ____
4. Delete your data after a certain amount of time. ____

Total ____

Base: All respondents

Q9_2. [Numeric] [Prompt once if responses do not equal 100]

Please allocate 100 points across the following protections. The more important a protection is to you, the more points it should receive.

**You can:**

*Scripter: display box for each option, with active sum in the Total row; randomize options*

1. Require companies to erase your data at any time. ____
2. Check that the data collected about you is accurate. ____
3. Opt-out of having your data collected. ____
4. Opt-out of having your data sold to third parties. ____

Total ____

Base: All respondents

Q9_3. [Numeric] [Prompt once if responses do not equal 100]

Please allocate 100 points across the following protections. The more important a protection is to you, the more points it should receive.

**The company will:**

*Scripter: display box for each option, with active sum in the Total row; randomize options*

1. Have an independent oversight board to make sure your data is protected. ____
2. Provide documentation that they are protecting your data. ____
3. Pay a fine to the government if they misuse or mishandle your data. ____

____

____

4. Pay you if they misuse or mishandle your data.
Total ___

Base: All respondents Q10. [S]

When you think about downloading apps in general, how concerned are you about your privacy?

1. Very concerned
2. Moderately concerned
3. Somewhat concerned
4. Slightly concerned
5. Not at all concerned

Base: All respondents Q11. [S]

In general, do you think of yourself as…

1. Extremely liberal
2. Liberal
3. Slightly liberal
4. Moderate, middle of the road
5. Slightly conservative
6. Conservative
7. Extremely conservative

**Show KP closing question QF1**
