# Supplementary material for: Consumer Confidence in the Responsible Use of Digital Health Data After the COVID-19 Pandemic
Source: JAMA Netw Open. 2025 Feb 26;8(2):e2461907. doi: 10.1001/jamanetworkopen.2024.61907 (PMC11866024; doi:10.1001/jamanetworkopen.2024.61907)
Supplement: Supplement 2. — Data Sharing Statement [file jamanetwopen-e2461907-s002.pdf]

Data Sharing Statement

Gupta. Consumer Confidence in the Responsible Use of Digital Health Data After the COVID-19 Pandemic. *JAMA Netw Open*. Published February 26, 2025.
doi:10.1001/jamanetworkopen.2024.61907

Data
Data available: No

Additional Information
Explanation for why data not available: Data will be made available upon request.
